# Efficacy and short-term safety of intravenous omadacycline as second-line therapy for macrolide- unresponsive Mycoplasma pneumoniae pneumonia in children: a retrospective cohort study

**DOI:** 10.3389/fcimb.2026.1863784

**Published:** 2026-07-22

**Authors:** Yang Yu, Tingting Zhang, Yu Xia, Yao Sun

**Affiliations:** Department of Pediatrics, Nanjing Lishui People’s Hospital, Zhongda Hospital Lishui Branch, Southeast University, Nanjing, China

**Keywords:** community-acquired pneumonia, macrolide resistance, macrolide-unresponsive pneumonia, Mycoplasma pneumoniae, omadacycline, pediatric pneumonia, tetracyclines

## Abstract

**Objective:**

To evaluate the efficacy and short-term safety of omadacycline as second-line therapy for macrolide-unresponsive Mycoplasma pneumoniae pneumonia (MUMPP) in children aged 8–16 years.

**Methods:**

A total of 64 hospitalized children with MUMPP admitted between September 2023 and December 2024 were enrolled. Children with persistent fever (>38.0 °C) after 72 hours of azithromycin monotherapy were assigned to receive intravenous omadacycline (n=18) or continued azithromycin (n=46). Omadacycline was dosed according to body surface area (BSA)-based scaling from the approved adult regimen (loading dose on day 1; maintenance dose once daily for 7 days). Primary outcome: time to defervescence (TTD). Secondary outcomes: 72-hour defervescence rate, radiological improvement, corticosteroid use, and adverse events. Three-month follow-up included dental examinations.

**Results:**

Omadacycline achieved shorter TTD than continued macrolides (50.78 ± 20.71 vs. 72.46 ± 25.61 hours; P = 0.002) and higher 72-hour defervescence rate (55.6% vs. 26.1%; P = 0.026). On multivariate linear regression, continued macrolide therapy (B = 13.43 h, 95% CI: 2.02–24.84, P = 0.022), positive 23S rRNA mutation status (B = 20.85 h, 95% CI: 10.94–30.77, P<0.001), and systemic corticosteroid use (B = 21.21 h, 95% CI: 10.79–31.62, P<0.001) were independently associated with prolonged TTD. No significant difference was observed in radiological improvement (P = 0.668). Corticosteroid requirement was significantly lower in the omadacycline group than in the macrolide group (27.8% vs. 67.4%; P = 0.004). Adverse events were comparable (5.6% vs. 8.7%; P = 0.645). At 3-month follow-up, no clinically significant changes were observed in hematological indices, hepatic or renal function, or cardiac enzyme profiles, and dental examination revealed no discoloration or enamel hypoplasia in any patient.

**Conclusion:**

In this retrospective cohort, omadacycline as second-line therapy was associated with significantly faster fever resolution than continued macrolide treatment, with acceptable short-term safety profiles over a 3-month follow-up. Prospective randomized trials are needed to confirm these findings.

## 1 Introduction

Mycoplasma pneumoniae (MP) is a major cause of community-acquired pneumonia (CAP) in school-age children and adolescents, accounting for 10–40% of pediatric CAP cases worldwide during epidemic periods (Upadhyay and Singh, 2024). Recurrent epidemics of MP infection continue to occur globally and impose a substantial burden on pediatric healthcare systems (Diaz et al., 2025). In recent years, both the incidence and the clinical severity of MP-associated pneumonia appear to have increased, with a notable post-pandemic resurgence reported in 2023 (Wu et al., 2024).

Macrolides have long been recommended as first-line therapy for pediatric Mycoplasma pneumoniae pneumonia (MPP) because of their favorable safety profile and intracellular activity (Lloyd and Wolf, 2025). However, their widespread and often empirical use has contributed to the rapid emergence of macrolide-resistant M. pneumoniae (MRMP), posing a major challenge to clinical management (Jia et al., 2024; Oishi et al., 2025). This problem is particularly pronounced in East Asia, where reported resistance rates exceed 80–90% (Yamazaki and Kenri, 2016; Yun, 2025). In M. pneumoniae, macrolide resistance is primarily mediated by point mutations in domain V of the 23S ribosomal RNA (rRNA) gene, especially A2063G and A2064G, which interfere with macrolide binding to the 50S ribosomal subunit (Zhu et al., 2024). As a result, an increasing proportion of children develop macrolide-unresponsive Mycoplasma pneumoniae pneumonia (MUMPP), which is characterized by persistent fever despite adequate macrolide therapy (Li et al., 2024; Liu et al., 2025).

The rising prevalence of MUMPP has created an urgent need for effective alternative antimicrobial strategies, and current guidelines recommend tetracyclines (e.g., doxycycline) or fluoroquinolones (e.g., levofloxacin) as second-line options (Wang et al., 2024). However, conventional tetracyclines have historically been used with caution in children because of concerns about tooth discoloration and potential effects on skeletal growth, whereas fluoroquinolones are associated with concerns regarding cartilage toxicity and arthropathy, thereby limiting their pediatric use (Li et al., 2022). Omadacycline is a novel aminomethylcycline antibiotic derived from the tetracycline class (Dougherty et al., 2019). It retains broad-spectrum activity against respiratory pathogens, including MRMP (Wu et al., 2025). Structural modifications at the C7 and C9 positions enhance its binding affinity for the 30S ribosomal subunit and may reduce calcium chelation, thereby theoretically mitigating the dental and skeletal risks associated with conventional tetracyclines (Duras and Sousa, 2019; Zhanel et al., 2020). In adults, omadacycline has demonstrated efficacy in community-acquired bacterial pneumonia (File et al., 2025; Wang et al., 2026). In addition, pediatric pharmacokinetic studies have shown predictable drug exposure after short-course administration, providing a pharmacological rationale for its use in children (Tanaka et al., 2016; Rodvold et al., 2020).

To date, evidence supporting the use of omadacycline in pediatric MUMPP is limited to isolated case reports (Xu and Fang, 2023), and no cohort-level studies have characterized its clinical efficacy, predictors of treatment response, or safety in school-age children. In high-resistance settings, where macrolide failure is common, this evidence gap leaves clinicians without a robust basis for treatment-escalation decisions. Moreover, safety data specific to children aged 8–16 years, in whom permanent dentition is still maturing, are essential before omadacycline can be more broadly considered in this population.

Because persistent fever is the cardinal clinical manifestation of MUMPP and a major driver of healthcare utilization and family distress, we selected time to defervescence (TTD) as the primary efficacy endpoint, as it represents a clinically meaningful and patient-centered outcome. We hypothesized that, in children with MUMPP who remained febrile despite azithromycin therapy, early escalation to omadacycline would result in faster fever resolution than continued macrolide therapy without causing clinically detectable dental or systemic toxicity.

## 2 Materials and methods

### 2.1 Study design and participants

This retrospective cohort study evaluated the clinical efficacy and short-term safety of intravenous omadacycline as second-line therapy in pediatric patients with macrolide-unresponsive Mycoplasma pneumoniae pneumonia (MUMPP), defined as MPP in children who, after receiving standard macrolide antibiotic therapy for 72 hours, continue to have persistent fever, with no improvement in clinical manifestations or pulmonary imaging findings, or whose condition further worsens (Wang et al., 2024). This study was reported in accordance with the Strengthening the Reporting of Observational Studies in Epidemiology (STROBE) guidelines for cohort studies. Consecutive pediatric inpatients aged 8–16 years admitted to the Department of Pediatrics at Lishui People’s Hospital, Nanjing, China, between September 2023 and December 2024 were screened for eligibility.

Patients were eligible for inclusion if they met all of the following criteria: (1) age 8–16 years at admission; (2) clinical features consistent with community-acquired pneumonia (CAP), including fever and acute respiratory symptoms [cough, tachypnea, or dyspnea] with pulmonary infiltrates or consolidation on chest imaging; (3) confirmed Mycoplasma infection, established through detection of Mycoplasma pneumoniae nucleic acid by polymerase chain reaction (PCR) in nasopharyngeal secretions — or, when PCR was negative, by serological criteria (paired sera) showing either (a) ≥4-fold rise in MP-IgG titer or (b) IgM seroconversion by chemiluminescent immunoassay (CLIA) — with single-serum IgM positivity alone not considered diagnostic; and (4) persistent fever (>38.0 °C) despite 72 hours of standard-dose azithromycin therapy (MUMPP). Patients were excluded if any of the following applied: (1) underlying chronic cardiopulmonary disease or immunodeficiency; (2) co-infection with another respiratory pathogen identified by comprehensive screening (sputum culture, blood culture, and multiplex qPCR for influenza, parainfluenza, RSV, coronavirus, rhinovirus, and adenovirus); (3) receipt of any non-macrolide antibiotic prior to or during the initial treatment period; or (4) incomplete clinical or follow-up data.

For MP-DNA–positive samples, M. pneumoniae nucleic acid and macrolide-resistance determinants were detected by targeted next-generation sequencing (Diminkang™ tNGS; DINFECTOME, Nanjing Difei Medical Technology, China). Following nucleic acid extraction from clinical specimens, this multiplex PCR-based assay enriches target regions—including MP-specific sequences and 23S rRNA mutations (A2063G, A2064G, A2067G, C2617G)—for high-throughput sequencing on a Class III NMPA-certified DIFSEQ-200 platform. Bioinformatic analysis encompassing quality filtering, reference mapping, and variant calling was performed according to the manufacturer’s protocol.

### 2.2 Treatment protocol

All enrolled patients received azithromycin (10 mg/kg once daily, intravenous infusion) as initial anti-mycoplasma therapy upon admission, with clinical response assessed daily by the attending medical team. Patients who remained febrile (>38.0 °C) after 72 hours of azithromycin treatment were classified as macrolide-unresponsive and eligible for second-line therapy. Second-line treatment allocation followed a two-step process. First, attending clinicians comprehensively evaluated each patient’s illness severity, radiographic progression, and trends in inflammatory markers to determine whether escalation to a non-macrolide agent was clinically warranted. Second, for patients deemed appropriate for omadacycline, the treatment option was discussed in detail with legal guardians. Given that omadacycline was a relatively novel agent with limited pediatric data at the time of this study, it was administered only after explicit written informed consent was obtained from legal guardians; when consent was declined, azithromycin therapy was continued. Patients were thereby allocated to either intravenous omadacycline (omadacycline group) or continued azithromycin (macrolide group). Because allocation depended on both clinician judgment and guardian consent rather than randomization, the resulting groups were not randomly assigned; the resulting potential for selection bias is addressed in the Limitations (Section 4.5). 23S rRNA resistance-mutation genotyping results were unavailable at all treatment-decision timepoints, and therefore resistance genotype did not influence allocation.

In the omadacycline group, intravenous omadacycline (Nuzyra^®^, Zhejiang Hisun Pharmaceutical Co., Ltd., Taizhou, China) was administered according to a dosing regimen derived from the approved adult intravenous regimen (loading dose: 200 mg on day 1; maintenance dose: 100 mg once daily) and converted to pediatric equivalents using body surface area (BSA) scaling. Because omadacycline (Nuzyra^®^) is not currently approved for pediatric use, its administration in this cohort constituted off-label use, undertaken in the context of the 2023–2024 M. pneumoniae epidemic, during which regional macrolide resistance exceeded 60% and evidence-based treatment-escalation protocols for macrolide-unresponsive children had not yet been formalized. As noted above, the off-label nature of the treatment, the BSA-based dosing approach, and the limited pediatric safety data available were explained in detail to legal guardians, from whom explicit written informed consent was obtained before treatment. Individual BSA was calculated using the Mosteller formula: BSA (m²) = √[height (cm) × weight (kg)/3600]. Pediatric doses were then determined as: pediatric dose = adult dose × (individual BSA/1.73 m²). In the macrolide group, azithromycin (intravenous infusion) therapy was continued for 7–10 days according to institutional practice, with the same dosage as the initial treatment. If patients in either group remained refractory after 72 hours of second-line therapy—evidenced by persistent fever alongside clinical or radiological worsening—systemic corticosteroids (intravenous methylprednisolone, 2 mg/kg/day for 3 days) were added as rescue treatment. All patients received standardized supportive care, including antipyretics, cough suppressants, fluid management, and supplemental oxygen when indicated. Vital signs and clinical symptoms were closely monitored throughout hospitalization, and adverse events were recorded in real time by nursing staff.

### 2.3 Data collection and outcome measures

Clinical data were extracted from electronic medical records using a standardized data collection form by two independent researchers, with discrepancies resolved by consensus or adjudication by a senior investigator. Collected variables included demographic characteristics, clinical manifestations, laboratory parameters, imaging findings, treatment details, and adverse events.

The primary outcome was time to defervescence (TTD), defined as the interval from initiation of second-line therapy to sustained normalization of body temperature (<37.3 °C for at least 24 consecutive hours). Secondary outcomes included: (1) the proportion of patients achieving defervescence within 72 hours of second-line therapy initiation; (2) radiological improvement (classified as marked improvement, partial improvement, or no improvement) at end of treatment; (3) requirement for systemic corticosteroids; and (4) incidence of adverse events.

Chest imaging was independently evaluated by two experienced pediatric radiologists blinded to treatment allocation. Inter-rater agreement was quantified using Cohen’s kappa coefficient; kappa values of 0.61–0.80 and >0.80 were considered substantial and almost perfect agreement, respectively. To assess short-term safety, all patients in the omadacycline group were scheduled for follow-up evaluations three months after treatment completion. Follow-up assessments included routine blood tests, liver and kidney function tests, cardiac enzyme profiles, and comprehensive dental examinations performed by dental specialists to detect tooth discoloration or enamel hypoplasia.

### 2.4 Statistical analysis

Statistical analyses were performed using SPSS version 26.0 (IBM Corp., Armonk, NY, USA). The normality of continuous variables was assessed using the Shapiro–Wilk test. Normally distributed data are presented as mean ± standard deviation (SD) and were compared between groups using the independent-samples t test; within-group comparisons between baseline and follow-up values were performed using the paired t test. Non-normally distributed data are presented as median (interquartile range, IQR) and were compared between groups using the Mann–Whitney U test; within-group pre- and post-treatment comparisons were performed using the Wilcoxon signed-rank test. Categorical variables are expressed as counts (percentages) and were compared between groups using the χ² test; when the expected cell counts were <5, the Fisher’s exact test was applied. Radiographic improvement, assessed as an ordinal three-category outcome (marked improvement, partial improvement, and no improvement), was compared between groups using the Mann–Whitney U test.

To evaluate factors associated with time to defervescence (TTD), univariate analyses were performed. For categorical variables with two groups, comparisons were conducted using the Student’s t test, whereas one-way analysis of variance (ANOVA) was used for categorical variables with three or more groups. Associations between continuous variables and TTD were assessed using Pearson’s correlation analysis. Variables with P < 0.10 in the univariate analysis, together with clinically relevant covariates, were entered into a multivariate linear regression model to identify independent predictors of TTD. Multicollinearity was evaluated using variance inflation factors (VIF), and the normality of model residuals was assessed using the Shapiro–Wilk test.

No imputation was performed for missing data; patients with incomplete records were excluded according to the eligibility criteria. All statistical tests were two-tailed, and a P value < 0.05 was considered statistically significant.

### 2.5 Ethical considerations

This study was approved by the Ethics Committee of Lishui People’s Hospital, Nanjing, Jiangsu Province, China (approval number: 2024KY08-02, granted August 2, 2024). Two distinct processes—clinical treatment and research participation—should be distinguished. With respect to clinical treatment, omadacycline was administered as part of routine clinical care, before this study was conceived as a research project. Because omadacycline is not approved for pediatric use, attending clinicians obtained explicit written informed consent for its off-label use from the legal guardians of every patient in the omadacycline group prior to treatment initiation, after explaining the off-label nature of the treatment, the BSA-based dosing approach, and the limited pediatric safety data then available. With respect to research participation, the study was conceived retrospectively, after these clinical events had occurred. Institutional review board approval was obtained before any research-specific activity—including retrospective record extraction, de-identification, and analysis—was undertaken. Because the analysis used only de-identified data generated during routine care and no additional data were collected for research purposes, the requirement for separate written informed consent for research participation was waived by the institutional review board in accordance with institutional data-protection policy. This sequence—in which research approval follows the clinical events under study—is intrinsic to the retrospective cohort design and does not reflect treatment administered for research purposes.

## 3 Results

### 3.1 Study population and baseline characteristics

**Figure 1:**
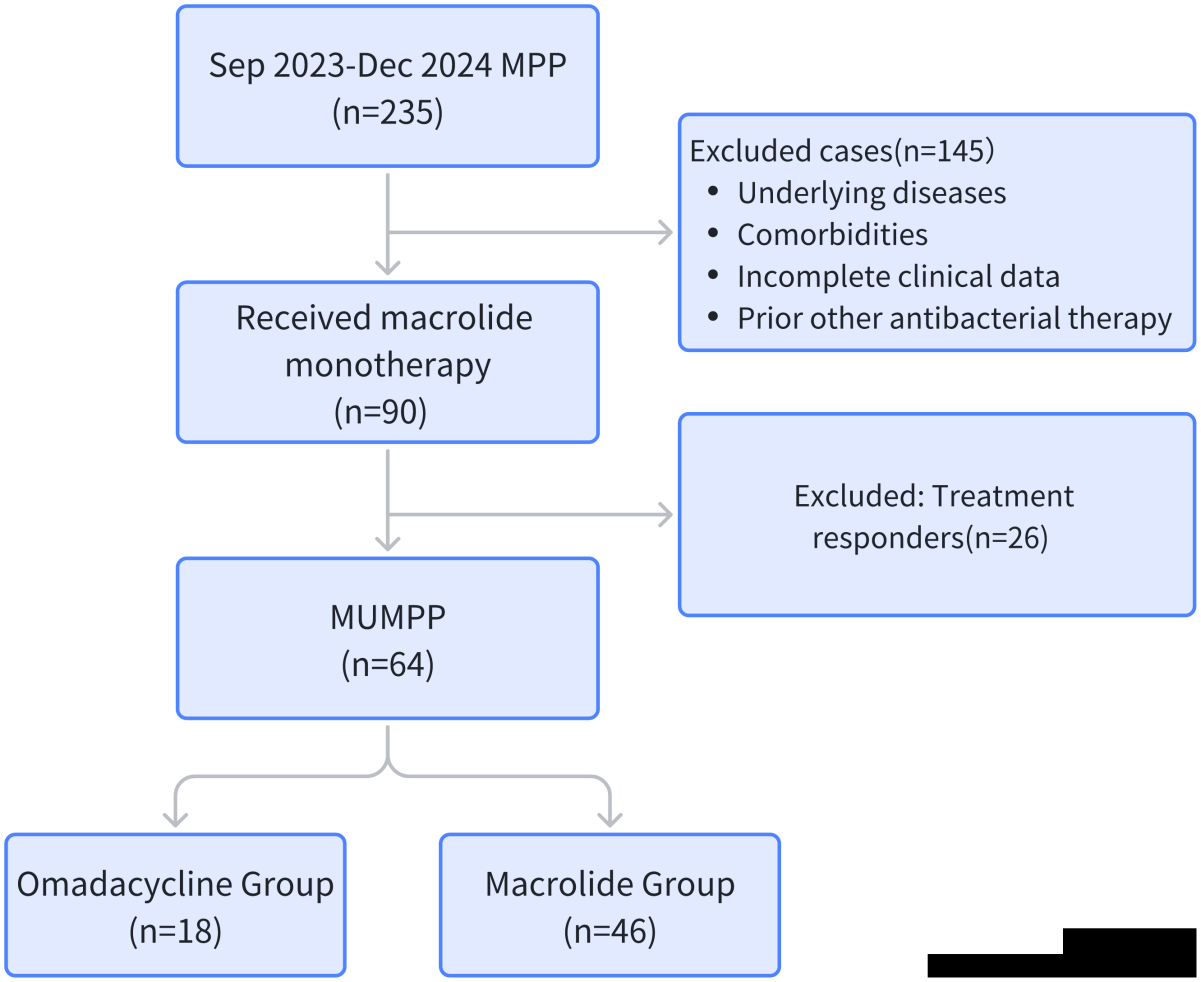
Flowchart for the study population. MPP, Mycoplasma pneumoniae pneumonia; MUMPP, macrolide-unresponsive mycoplasma pneumoniae pneumonia.

Between September 2023 and December 2024, a total of 235 hospitalized children aged ≥8 years with community-acquired MPP were screened for eligibility. After exclusion of patients with underlying chronic diseases, co-infections, incomplete clinical data, or prior exposure to non-macrolide antibiotics, 90 patients who received initial macrolide therapy were included. Among them, 26 patients responded to macrolide treatment and were excluded. The remaining 64 patients met the predefined criteria for MUMPP and constituted the final study cohort (**Figure 1**).

Of these 64 patients, 18 were switched to omadacycline after 72 hours of initial macrolide therapy (omadacycline group), and 46 continued azithromycin therapy (macrolide group). Baseline demographic characteristics, clinical manifestations, radiological findings, and laboratory parameters were comparable between the two groups, with no statistically significant differences observed (all P > 0.05; **Table 1**). The positivity rate of the resistance gene was 61.1% (11/18) in the omadacycline group and 63.0% (29/46) in the macrolide group, with no significant difference between groups (χ² = 0.021, P = 0.887; OR = 1.086, 95% CI 0.354–3.330; **Table 1**).

**Table 1:** Baseline demographic, clinical, and laboratory characteristics of children with MUMPP.

Variable	OMC (n=18)	ML (n=46)	t/χ²/z	P-value
Age, years	11.5 (9.0–13.0)	11.0 (9.0–13.75)	−0.233	0.815
Sex, male, n (%)	8 (44.4)	24 (52.2)	0.309	0.578
BMI, kg/m²	18.13 ± 1.89	17.76 ± 2.32	0.663	0.511
Temperature, °C	38.74 ± 0.60	38.81 ± 0.56		0.698
SaO_2_, %	94.80 (93.28–96.15)	95.55 (93.70–97.78)	−1.06	0.292
Extrapulmonary complications, n (%)	6 (33.3)	8 (17.4)	Fisher	0.191
Fever duration pre-treatment, days	5.0 (3.0–6.0)	5.5 (4.0–6.0)	−1.327	0.158
Chest imaging, n (%)			0.787	0.675
Patchy infiltration	5 (27.8)	12 (26.1)		
Focal consolidation	7 (38.9)	23 (50.0)		
Lobar consolidation	6 (33.3)	11 (23.9)		
23S rRNA mutation positive, n (%)	11 (61.1)	29 (63.0)	0.021	0.887
WBC, ×10^9^/L	8.53 (7.23–11.90)	8.04 (6.28–10.16)	−1.09	0.276
CRP, mg/L	7.22 (4.11–19.56)	11.58 (3.92–17.01)	−0.672	0.502
Neutrophils, ×10^9^/L	4.84 (3.88–7.43)	5.04 (3.53–6.37)	−0.015	0.988
D-dimer, ng/mL	447.50 (325.00–620.00)	391.50 (253.00–495.00)	−1.38	0.165
LDH, U/L	295.50 (263.0–329.00)	291.50 (246.00–315.00)	−1.038	0.299
ALT, U/L	23.96 ± 1.65	24.05 ± 1.43	0.229	0.252
AST, U/L	36.12 ± 1.87	37.17 ± 1.67	0.123	0.379
BUN, mmol/L	3.90 ± 0.10	3.95 ± 0.09	−0.54	0.590

Values are presented as median (interquartile range), mean ± SD, or n (%), as appropriate.

MUMPP, macrolide-unresponsive Mycoplasma pneumoniae pneumonia; OMC, omadacycline group; ML, macrolide group; BMI, body mass index; SaO_2_, arterial oxygen saturation; WBC, white blood cell count; CRP, C-reactive protein; LDH, lactate dehydrogenase; ALT, alanine transaminase; AST, aspartate aminotransferase; BUN, blood urea nitrogen.

### 3.2 Comparison of clinical outcomes

Patients in the omadacycline group achieved significantly faster fever resolution than those in the macrolide group (mean TTD: 50.78 ± 20.71 h vs. 72.46 ± 25.61 h; mean difference: −21.7 h, 95% CI: −35.4 to −7.9 h; P = 0.002). Consistent with this finding, Kaplan–Meier analysis demonstrated a significant difference between groups (log-rank P = 0.006). The proportion of patients achieving defervescence within 72 h was higher in the omadacycline group than in the macrolide group (55.6% [10/18] vs. 26.1% [12/46]; OR = 3.542, 95% CI: 1.134–11.06; χ²=4.99, P = 0.026). No significant difference was observed between groups in radiological improvement (Mann–Whitney U = 387, Z=−0.429, P = 0.668). The proportion of corticosteroid use was significantly lower in the omadacycline group than in the macrolide group (27.8% [5/18] vs. 67.4% [31/46]; OR = 5.37, 95% CI: 1.616–17.868; χ²=8.250, P = 0.004) (**Table 2**).

**Table 2:** Comparison of clinical outcomes between omadacycline and macrolide therapy.

Outcome	OMC (n=18)	ML (n=46)	Statistic	P-value
Time to defervescence (TTD), h	50.78 ± 20.71	72.46 ± 25.61	t = −3.20	0.002
Defervescence within 72 h, n (%)	10 (55.6)	12 (26.1)	χ² = 4.99	0.026
Systemic corticosteroid use, n (%)	5 (27.8)	31 (67.4)	χ² = 8.25	0.004
Radiographic improvement, n (%)			Z = −0.429	0.668
Marked improvement	7 (38.9)	17 (37.0)		
Partial improvement	7 (38.9)	15 (32.6)		
No improvement	4 (22.2)	14 (30.4)		

Values are presented as mean ± SD or n (%). Continuous variables were compared using Student’s t-test. Categorical variables were compared using the χ² test or Fisher’s exact test, as appropriate. Radiographic improvement was assessed as an ordinal outcome and compared using the Mann–Whitney U test.

OMC, omadacycline group; ML, macrolide group; TTD, time to defervescence.

### 3.3 Factors associated with time to defervescence

Univariate analyses identified three variables significantly associated with TTD (**Table 3**). Patients treated with omadacycline had a significantly shorter TTD than those who continued macrolide therapy (50.78 ± 20.71 vs. 72.46 ± 25.61 h; t = −3.20, P = 0.002). Positive 23S rRNA mutation status was associated with a longer TTD compared with negative mutation status (75.10 ± 24.70 vs. 51.79 ± 21.79 h; t = −3.81, P < 0.001). Likewise, patients who received systemic corticosteroids had a prolonged TTD relative to those who did not (77.67 ± 23.12 vs. 51.82 ± 22.46 h; t = −4.49, P < 0.001). No significant associations with TTD were found for sex, chest imaging pattern, age, body mass index, fever duration before treatment, arterial oxygen saturation, white blood cell count, C-reactive protein, D-dimer, or lactate dehydrogenase. Although age showed a borderline association with TTD (P = 0.070), it did not reach statistical significance.

**Table 3:** Univariate analysis of factors associated with time to defervescence (TTD).

Variable	Category/statistic	TTD (h)	t/F/r	P-value
Drug				
Omadacycline (OMC)		50.78 ± 20.71		
Macrolides (ML)		72.46 ± 25.61	t = −3.20	0.002
23S rRNA mutation				
Negative		51.79 ± 21.79		
Positive		75.10 ± 24.70	t = −3.81	<0.001
Corticosteroid use				
No		51.82 ± 22.46		
Yes		77.67 ± 23.12	t = −4.49	<0.001
Sex				
Male		70.59 ± 25.59		
Female		62.12 ± 26.28	t = −1.31	0.195
Chest imaging				
Patchy bilateral infiltrates		57.52 ± 18.20		
Focal consolidation		68.66 ± 24.81		
Lobar consolidation		71.71 ± 33.41	F = 1.351	0.257
Age, years	r = 0.234		—	0.070
BMI, kg/m²	r = −0.032		—	0.802
Fever duration pre-treatment, days	r = 0.145		—	0.253
SaO_2_, %	r = −0.148		—	0.242
WBC, ×10^9^/L	r = 0.076		—	0.551
CRP, mg/L	r = 0.037		—	0.774
D-dimer, ng/mL	r = 0.162		—	0.200
LDH, U/L	r = −0.146		—	0.250

Values are presented as mean ± standard deviation unless otherwise indicated. Comparisons between two groups were performed using Student’s t-test; comparisons among chest imaging categories were conducted using one-way ANOVA; correlations between continuous variables and TTD were assessed using Pearson’s correlation analysis (r).

TTD, time to defervescence; BMI, body mass index; SaO_2_, arterial oxygen saturation; WBC, white blood cell count; CRP, C-reactive protein; LDH, lactate dehydrogenase.

Variables with P < 0.10 in univariate analyses, together with chest imaging pattern as a pre-specified clinically relevant covariate, were entered into a multivariate linear regression model. After adjustment, continued macrolide therapy, positive 23S rRNA mutation status, and systemic corticosteroid use remained independently associated with prolonged TTD. Compared with omadacycline therapy, continued macrolide treatment was associated with a 13.43-hour increase in TTD (95% CI: 2.02–24.84; P = 0.022). Positive 23S rRNA mutation status and systemic corticosteroid use were associated with increases of 20.85 hours (95% CI: 10.94–30.77; P < 0.001) and 21.21 hours (95% CI: 10.79–31.62; P < 0.001), respectively. Among chest imaging categories, lobar pneumonia was independently associated with longer TTD than patchy bilateral infiltrates (B = 18.12 hours, 95% CI: 4.94–31.30; P = 0.008), whereas focal consolidation showed no significant association (B = 10.39 hours, 95% CI: −1.27 to 22.06; P = 0.080). The overall model was statistically significant (F = 12.083, P < 0.001), explaining 51.0% of the variance in TTD (R² = 0.510; adjusted R² = 0.468). All variance inflation factors were <2, indicating no evidence of multicollinearity. Residuals were approximately normally distributed based on the Shapiro–Wilk test (W = 0.978, P = 0.321) (**Table 4**).

**Table 4:** Multivariate linear regression analysis of factors associated with time to defervescence (TTD).

Variable	B (hours)	95% CI	Standardized β	P-value	VIF
Drug (ML vs. OMC)a	13.43	2.02 – 24.84	0.233	0.022	1.161
23S rRNA mutation (positive vs. negative)a	20.85	10.94 – 30.77	0.390	<0.001	1.017
Steroid use (yes vs. no)a	21.21	10.79 – 31.62	0.406	<0.001	1.178
Chest imaging (focal consolidation vs. patchy)b	10.39	−1.27 – 22.06	0.200	0.080	1.495
Chest imaging (lobar vs. patchy)b	18.12	4.94 – 31.30	0.309	0.008	1.494

TTD is defined as the time from second-line therapy initiation to sustained defervescence (temperature <37.3 °C for ≥24 h).

^a^ Dummy coding: Drug (reference = OMC); 23S rRNA mutation (reference = negative); steroid use (reference = no).

^b^ Reference category for chest imaging = patchy bilateral infiltrates.

OMC, omadacycline group; ML, macrolide group; CI, confidence interval; VIF, variance inflation factor.

### 3.4 Safety outcomes

Adverse events were infrequent in both groups. In the omadacycline group, one patient (5.6%) experienced mild gastrointestinal symptoms; no cases of rash or tooth discoloration were observed. Four patients (8.7%) in the macrolide group reported adverse events (**Table 5**). The overall incidence of adverse events did not differ significantly between groups (χ² = 0.21, P = 0.645). No severe adverse events or treatment discontinuations occurred in either group. No abnormalities in liver or kidney function tests were detected during hospitalization in the omadacycline group.

**Table 5:** Comparison of adverse events between the two treatment groups.

Group	n	Digestive system symptoms	Rash	Tooth discoloration	Total adverse reactions
OMC	18	1 (5.6)	0 (0)	0 (0)	1 (5.6)
ML	46	3 (6.5)	1 (2.2)	0 (0)	4 (8.7)
χ² value					0.21
P-value					0.645

Data are presented as n (%). The χ² test was used to compare the total incidence of adverse events between groups.

OMC, omadacycline group; ML, macrolide group.

### 3.5 Follow-up outcomes

All 18 patients in the omadacycline group completed the three-month follow-up. No significant changes in hematological indices, liver or kidney function, or cardiac enzyme profiles were observed compared with baseline (all P > 0.05; **Table 6**). Dental examinations demonstrated normal tooth development in all patients, with no evidence of tooth discoloration or enamel hypoplasia.

**Table 6:** Three-month follow-up laboratory and dental outcomes in the omadacycline group.

Parameter	Baseline	3-month follow-up	P-value
WBC (×10^9^/L)	8.53 (7.23–11.90)	8.11 (6.84–9.75)	0.548
Hb (g/L)	128 (120–136)	129 (121–137)	0.782
PLT (×10^9^/L)	285 (245–320)	279 (238–308)	0.713
ALT (U/L)	22 (18–27)	23 (19–28)	0.694
AST (U/L)	38 (33–43)	37 (31–42)	0.768
BUN (mmol/L)	3.9 (3.6–4.2)	4.0 (3.7–4.3)	0.433
Scr (μmol/L)	62.5 (56.0–69.0)	63.1 (57.0–69.5)	0.802
CK (U/L)	65 (48–82)	62 (45–79)	0.615
CK-MB (U/L)	12 (8–16)	11 (7–15)	0.583
Dental health evaluation	Normal (100.0%)	Normal (100.0%)	—

Data are presented as median (interquartile range). Paired comparisons were performed using the Wilcoxon signed-rank test or paired t-test, as appropriate. Dental health was evaluated by specialist examination.

WBC, white blood cell count; Hb, hemoglobin; PLT, platelet; ALT, alanine transaminase; AST, aspartate aminotransferase; BUN, blood urea nitrogen; Scr, serum creatinine; CK, creatine kinase; CK-MB, creatine kinase-MB isoenzyme.

## 4 Discussion

### 4.1 Principal findings

In this retrospective cohort of children aged 8–16 years with macrolide-unresponsive *Mycoplasma pneumoniae* pneumonia (MUMPP), switching to intravenous omadacycline resulted in a clinically meaningful 22-hour reduction in time to defervescence compared with continued azithromycin therapy (50.8 vs. 72.5 hours; P = 0.002), with 55.6% achieving defervescence within 72 hours versus 26.1% in the macrolide group. Omadacycline was additionally associated with a substantially lower requirement for systemic corticosteroids (27.8% vs. 67.4%; P = 0.004), whereas no significant between-group difference was observed in radiological improvement. Three-month follow-up revealed no detectable hematological, hepatic, renal, cardiac, or dental abnormalities. Multivariate analysis identified four independent predictors of prolonged TTD — continued macrolide therapy, 23S rRNA mutation positivity, systemic corticosteroid use, and lobar consolidation — explaining 51.0% of the variance in fever duration and providing a quantitative basis for individualized risk stratification.

These findings make three distinct contributions to the existing literature. First, this study provides, to our knowledge, the first cohort-level efficacy and safety data for intravenous omadacycline as second-line therapy in school-age children with MUMPP — a population for whom the only prior evidence consisted of isolated case reports, leaving clinicians without a real-world evidence base for treatment-escalation decisions. Second, by directly linking individual-level 23S rRNA genotyping data to clinical fever trajectories within a single analytic framework, this study moves beyond descriptive resistance prevalence reporting to establish molecular resistance as a quantifiable, independent determinant of treatment response — an approach not previously applied in pediatric MUMPP cohort studies. Third, the identification of lobar consolidation as an independent predictor of prolonged TTD after multivariate adjustment — a finding that was invisible in unadjusted analysis owing to confounding masking — demonstrates the added inferential value of multivariate modeling in this clinical context and provides a novel imaging-based parameter for early risk stratification.

### 4.2 Interpretation of key findings

#### 4.2.1 Efficacy of omadacycline in the context of high macrolide resistance

Macrolide-resistant M. pneumoniae (MRMP) has become the dominant strain in many regions, with resistance rates exceeding 80–90% in East Asia (Wang and Tu, 2024; Yan et al., 2026). This resistance stems mainly from point mutations in domain V of the 23S rRNA gene (A2063G and A2064G), which reduce macrolide binding to the 50S ribosomal subunit. In our cohort, 61.1% of patients in the omadacycline group and 63.0% in the macrolide group carried these mutations (P = 0.887), confirming that MRMP was highly prevalent and that the two groups were comparable in this regard.

The 22-hour reduction in time to defervescence (TTD) with omadacycline fits its mechanism of action. Unlike macrolides, omadacycline binds to the 30S ribosomal subunit at a non-overlapping site, so its activity is preserved against MRMP regardless of 23S rRNA mutation status. Its C9-aminomethyl modification also improves ribosomal binding and provides protection against tetracycline efflux pumps and ribosomal protection proteins (TetO/TetM) (Mendes et al., 2020). These properties differentiate it from doxycycline, which remains vulnerable to ribosomal protection-mediated resistance in some MRMP strains (Waites et al., 2016).

Because genotyping was not available when treatment decisions were made, allocation was effectively independent of resistance status. The similar mutation prevalence between groups makes it unlikely that the TTD difference was driven by uneven strain distribution. More importantly, linking *post-hoc* genotyping to individual outcomes allowed us to isolate the effect of molecular resistance on fever duration. We found that 23S rRNA mutation positivity independently prolonged TTD by 20.85 hours (95% CI: 10.94–30.77; P < 0.001). To our knowledge, this is the first cohort-level quantification of this association in pediatric MUMPP. It indicates that genotyping can serve not merely as an epidemiological tool, but as a practical predictor to guide early non-macrolide therapy instead of waiting for clinical failure.

Chest imaging pattern was not associated with TTD in univariate analysis (F = 1.351, P = 0.257) but was retained in the multivariate model as a pre-specified covariate. After adjustment for treatment, resistance status, and corticosteroid use, lobar consolidation predicted longer TTD than patchy infiltration (B = 18.12 hours, 95% CI: 4.94–31.30; P = 0.008), whereas focal consolidation did not (B = 10.39 hours, 95% CI: −1.27 to 22.06; P = 0.080). That lobar consolidation only emerged as significant after adjustment suggests its effect was masked by confounding—likely because patients with more severe radiological findings were also more likely to receive corticosteroids or be assigned to the macrolide group. This illustrates a broader point: radiological severity may appear uninformative in unadjusted analyses of MUMPP precisely when it matters most. For clinicians, it means that lobar consolidation on admission imaging is a readily available marker for risk stratification, requiring no specialized testing.

#### 4.2.2 Corticosteroid-sparing effect and radiological discordance

In contrast to the macrolide group, the omadacycline group had a significantly lower rate of systemic corticosteroid use (27.8% vs. 67.4%; χ² = 8.25, P = 0.004). This difference most plausibly reflects the more rapid fever resolution achieved with omadacycline: as corticosteroids in our protocol were reserved for patients with persistent fever accompanied by clinical deterioration or radiological progression within 72 hours of second-line therapy initiation, earlier defervescence in the omadacycline group would naturally reduce the proportion meeting this escalation threshold.

This corticosteroid-sparing effect represents a clinically meaningful dimension of omadacycline’s therapeutic value that extends beyond its primary antibacterial activity. In the context of pediatric MUMPP, where systemic corticosteroids — while effective for symptom relief — carry risks including immunosuppression, hyperglycemia, and growth effects with repeated courses, a treatment strategy that achieves comparable or superior clinical outcomes while substantially reducing steroid exposure addresses a long-standing unmet need in this population. Importantly, this effect appears to be mechanistically driven rather than coincidental: the causal pathway from faster bacterial clearance to earlier defervescence to reduced corticosteroid requirement constitutes a coherent biological cascade that, if confirmed in prospective randomized trials, would position omadacycline not merely as an antibiotic alternative but as a steroid-sparing strategy — a therapeutic framing with substantial implications for guideline development and treatment algorithm design.

Notably, despite the lower corticosteroid use in the omadacycline group, systemic corticosteroid administration remained an independent predictor of prolonged TTD in multivariate analysis (B = 21.21 hours, 95% CI: 10.79–31.62; P < 0.001), even after adjustment for treatment group and 23S rRNA mutation status. This finding most likely reflects confounding by indication: patients who received corticosteroids were inherently those with more severe or refractory disease, which independently prolonged fever duration regardless of antibiotic assignment. A direct immunomodulatory effect — whereby corticosteroid-mediated suppression of the inflammatory response transiently delays fever resolution — cannot be excluded, but cannot be distinguished from confounding in the present design.

No significant between-group difference was observed in radiological improvement at end of treatment (P = 0.668), consistent with the well-recognized discrepancy between clinical symptom resolution and radiological recovery in *Mycoplasma pneumoniae* pneumonia (Huang et al., 2024).Pulmonary infiltrates frequently persist beyond clinical improvement, and the anti-inflammatory effects of corticosteroids may independently accelerate radiological resolution in a subset of patients, thereby attenuating the ability to detect antibiotic-related differences over the short observation window of this study. In prospective cohorts of refractory MPP, the median time to complete radiographic clearance has been reported as 4 weeks, with 27% of patients requiring >8 weeks for resolution despite earlier clinical improvement (Huang et al., 2018). Collectively, these findings support the use of fever resolution — rather than radiological clearance — as the more responsive and clinically meaningful primary endpoint for evaluating antibiotic efficacy in pediatric MUMPP.

#### 4.2.3 Comparison with established second-line agents

Doxycycline and levofloxacin currently represent the most widely recommended second-line agents for pediatric MUMPP, and contextualizing the efficacy of omadacycline against this existing therapeutic landscape is essential for assessing its potential clinical role. It should be noted at the outset that the present study did not include a direct head-to-head comparison between omadacycline and these established agents; the following discussion therefore draws on indirect comparisons with recently published evidence and should be interpreted accordingly.

Recent real-world studies provide important benchmarks for evaluating second-line agent performance in pediatric MUMPP. In a retrospective cohort of 382 children, oral doxycycline (2 mg/kg twice daily) significantly outperformed azithromycin combined with methylprednisolone across multiple endpoints, including shorter hospital stay (median 6 vs. 8 days), lower rates of refractory MPP (4.3% vs. 14.3%), and higher radiological improvement rates (94.3% vs. 77.1%), with no evidence of tooth discoloration at 3-month follow-up. Consistent with these findings, a prospective propensity score-matched analysis demonstrated that both doxycycline and levofloxacin achieved comparably rapid defervescence (median 0 days for both) and were significantly superior to continued azithromycin (median 3 days), with no musculoskeletal complications or serious adverse events reported in either group (Ha et al., 2018). Collectively, these data establish that both doxycycline and levofloxacin confer meaningful clinical benefit over continued macrolide therapy, providing an important comparative reference for the present findings (Ha et al., 2018; Song et al., 2024; Yang et al., 2025).

Against this backdrop, the approximately 22-hour reduction in TTD observed with omadacycline in the present study appears clinically meaningful, although direct efficacy comparisons with doxycycline or levofloxacin cannot be made from the available data. Critically, however, the present study addresses a therapeutic niche that existing comparative data for doxycycline and levofloxacin do not fully cover. Neither doxycycline nor levofloxacin trials in pediatric MUMPP have systematically integrated molecular resistance genotyping with individual-level outcome data; thus, whether their reported efficacy is maintained specifically in 23S rRNA mutation-positive patients — rather than the broader MUMPP population — remains unknown. By contrast, the present study demonstrates that omadacycline achieves clinically meaningful fever resolution irrespective of 23S rRNA mutation status, with mutation positivity independently prolonging TTD by approximately 21 hours regardless of antibiotic assignment, implying that omadacycline’s mechanism of action is not compromised by the dominant resistance determinants in this population. This genotype-stratified efficacy profile represents a pharmacological advantage over doxycycline — whose susceptibility to ribosomal protection proteins (TetO/TetM) in a subset of MRMP strains introduces uncertainty about its activity at the molecular level — and over levofloxacin, which, despite its broad activity, carries cartilage toxicity concerns that restrict its use to cases where first- and second-line tetracyclines have failed (Bradley et al., 2014; Patel and Goldman, 2016).

From a pharmacological standpoint, several features of omadacycline may confer distinct advantages within this therapeutic class. First, the C9-aminomethyl modification enhances ribosomal binding affinity and confers resistance to both tetracycline efflux pump mechanisms and ribosomal protection proteins (TetO/TetM), providing broader activity against tetracycline-resistant strains compared with doxycycline (Song et al., 2024). Second, omadacycline offers once-daily intravenous dosing, in contrast to the twice-daily regimen required for doxycycline, which may improve treatment adherence and simplify inpatient management (Rodvold et al., 2020). Third, whereas fluoroquinolones carry well-documented concerns regarding cartilage toxicity and arthropathy that limit their routine use in children (Bradley et al., 2014; Patel and Goldman, 2016), omadacycline has demonstrated a favorable musculoskeletal safety profile in available adult and pediatric data. Fourth, the C9-aminomethyl structural modification may reduce calcium chelation relative to conventional tetracyclines, potentially attenuating the risk of dental and skeletal toxicity — a consideration of particular importance in the 8–16year age group, in whom permanent tooth mineralization may still be ongoing (Kim et al., 2022).

In current clinical practice, the selection between doxycycline and levofloxacin is itself subject to important constraints. Fluoroquinolones are generally reserved for cases in which tetracyclines are contraindicated or have failed, and specific evidence supports levofloxacin’s role in corticosteroid-resistant refractory MUMPP where other second-line options have been exhausted (Ha et al., 2018; Yang et al., 2025). While omadacycline cannot yet be positioned as a first-choice second-line agent in the absence of direct comparative randomized trial data, the present findings support its consideration as an evidence-based alternative for school-age children with MUMPP who fail initial macrolide therapy — particularly in high-resistance clinical settings where doxycycline may be unavailable, where levofloxacin is contraindicated, or where once-daily intravenous administration offers a practical management advantage. Prospective head-to-head trials are needed to formally establish omadacycline’s place within the pediatric MUMPP treatment algorithm.

An important consideration in interpreting the present findings is the choice of continued azithromycin as the comparator. While current guidelines recommend doxycycline or fluoroquinolones as second-line therapy for macrolide-unresponsive M. pneumoniae pneumonia, the continued use of azithromycin in the comparator group reflected the prevailing clinical practice at our institution during the 2023–2024 epidemic period, when tetracycline-class antibiotics were used with considerable caution in children due to longstanding safety concerns, fluoroquinolone use was restricted, and formalized treatment-escalation pathways had not yet been established. Continued azithromycin therefore represented the pragmatically relevant real-world comparator for this retrospective cohort study. However, this comparator choice means that the efficacy advantage observed for omadacycline cannot be extrapolated to superiority over doxycycline or levofloxacin, and head-to-head randomized trials against these established agents are essential to define omadacycline’s relative position within the pediatric MUMPP treatment algorithm.

### 4.3 Safety and tolerability

The safety of tetracycline-class antibiotics in pediatric populations has historically been a source of concern, particularly with respect to tooth discoloration and potential adverse effects on skeletal development (Warmling et al., 2024). In the present study, omadacycline demonstrated a favorable tolerability profile during the treatment period, with only one patient (5.6%) experiencing mild gastrointestinal symptoms — a rate comparable to that observed in the macrolide group (8.7%; *P* = 0.645). No cases of tooth discoloration, enamel hypoplasia, rash, or clinically significant systemic toxicity were identified during hospitalization or at the three-month follow-up assessment. The absence of hepatic, renal, hematological, or cardiac abnormalities at follow-up, with all paired laboratory parameters remaining stable relative to baseline (all *P* > 0.05), provides additional reassurance regarding the short-term organ-level safety of this regimen.

These findings are consistent with and contextualized by emerging epidemiological evidence on tetracycline-associated dental toxicity. A large-scale population-based study demonstrated that short-course tetracycline therapy (fewer than 14 days) in school-age children is associated with a markedly lower risk of dental discoloration than previously assumed, with a 5-year cumulative incidence of only 0.8% in the 8–16year age group compared with 4.1% in younger children (Kim et al., 2022). In the present cohort, three biologically convergent factors act in concert to further attenuate dental risk below even this low baseline. First, omadacycline’s C9-aminomethyl modification reduces calcium chelation affinity relative to earlier tetracyclines — the primary mechanism by which tetracyclines incorporate into mineralizing enamel — thereby reducing the pharmacodynamic driver of dental discoloration at the molecular level (Warner et al., 2022). Second, the age distribution of the present cohort (8–16 years) corresponds to a developmental window during which permanent tooth mineralization is largely complete for most dentition, substantially reducing the pool of susceptible enamel matrices available for drug incorporation (Tan et al., 2025). Third, the seven-day treatment course employed in this study minimizes cumulative drug exposure, further attenuating risk proportionally. The convergence of these three independent risk-mitigating mechanisms — structural, developmental, and temporal — provides a multi-layered biological rationale for why clinically detectable dental toxicity was absent in this cohort, and supports the conclusion that the historical safety concerns surrounding tetracycline use in children are not straightforwardly applicable to short-course omadacycline in this specific age group. To our knowledge, this study provides the most systematically characterized short-term dental safety dataset for any tetracycline-class antibiotic in children aged 8–16 years with respiratory infection to date, incorporating specialist dental examination at three-month follow-up across all treated patients.

From a pharmacokinetic perspective, omadacycline’s favorable tissue penetration and predictable drug exposure profiles following intravenous administration in pediatric populations provide additional support for its safety and therapeutic adequacy in this age group (Tanaka et al., 2016). It is nonetheless important to emphasize that the safety data reported here pertain exclusively to short-term intravenous administration and cannot be extrapolated to prolonged, repeated, or oral courses. The small sample size of the omadacycline group (n = 18) also limits the statistical power to detect rare adverse events. Nevertheless, the consistent absence of detectable toxicity across multiple organ systems and the three-month dental surveillance data collectively provide preliminary but limited evidence supporting the cautious short-term use of omadacycline in carefully selected children aged 8–16 years with MUMPP. It should be emphasized that the small sample size of the omadacycline group (n = 18) provides insufficient statistical power to detect rare adverse events (e.g., events with an incidence below 5%), and the three-month follow-up period, while adequate for detecting early post-treatment toxicity, is insufficient to assess delayed dental effects or long-term musculoskeletal safety. These safety findings should therefore be regarded as hypothesis-generating rather than definitive.

### 4.4 Clinical implications

This study provides real-world clinical evidence to inform treatment decision-making in a pediatric population for whom effective and safe second-line therapeutic options remain limited. To our knowledge, this represents one of the first cohort-level investigations to systematically evaluate intravenous omadacycline as second-line therapy for MUMPP in school-age children, integrating clinical efficacy outcomes, molecular resistance characterization, and structured short-term multi-organ safety surveillance within a single study.

The findings carry several practical implications for clinical management. In routine practice settings where molecular resistance testing is frequently delayed or unavailable, the present data suggest that omadacycline may serve as a pragmatic and effective escalation option for children aged 8–16 years who remain febrile after 72 hours of azithromycin therapy, particularly in regions with documented high MRMP prevalence. The approximately 22-hour reduction in TTD observed in the omadacycline group, if confirmed in larger prospective studies, may translate into clinically meaningful benefits including shorter hospitalization, reduced healthcare resource utilization, and diminished patient and caregiver burden — although these downstream outcomes were not directly assessed in the present study and await prospective evaluation. Furthermore, the significantly lower corticosteroid requirement observed in the omadacycline group (27.8% vs. 67.4%; *P* = 0.004) may represent an additional clinical benefit, potentially reducing patients’ exposure to steroid-related adverse effects, though the causal interpretation of this finding requires confirmation in a randomized design.

Beyond its immediate therapeutic implications, this study contributes to a growing mechanistic understanding of treatment response heterogeneity in pediatric MUMPP. The multivariate regression model, which explained 51.0% of the variance in TTD (adjusted R² = 0.468), identified four independent predictors of prolonged fever duration — continued macrolide therapy, 23S rRNA mutation positivity, systemic corticosteroid use, and lobar consolidation (Kang et al., 2025). Translated into clinical practice, these four predictors define a risk stratification framework with immediate bedside applicability: a child presenting with lobar consolidation on chest imaging, a positive 23S rRNA mutation result (where available), or early clinical deterioration within 72 hours of macrolide initiation represents a high-risk profile in whom prompt escalation to omadacycline is most likely to yield clinically meaningful benefit. This framework shifts the clinical management of MUMPP from a reactive, time-dependent response to macrolide failure toward a proactive, biomarker-informed stratification approach — a paradigm that, if validated prospectively, could substantially reduce the time-to-escalation and thereby shorten the period of unnecessary antibiotic continuation in children unlikely to respond to macrolides. The practical significance of this shift is amplified in the context of resource-limited settings where molecular resistance testing remains inaccessible: lobar consolidation, detectable by routine chest radiography, provides a universally available imaging surrogate that can flag high-risk patients for early escalation without requiring genotypic data.

### 4.5 Strengths, limitations, and future directions

This study makes four discrete contributions to the evidence base for pediatric MUMPP management. First, it provides what is, to our knowledge, the first cohort-level efficacy and safety evidence for intravenous omadacycline as second-line therapy specifically in school-age children aged 8–16 years with MUMPP — a population for whom the prior literature consisted exclusively of isolated case reports, leaving a critical evidence vacuum in a high-resistance clinical context where treatment-escalation decisions have historically been made without real-world pediatric data. Second, by coupling individual-level 23S rRNA genotyping with prospective clinical outcome tracking, this study establishes — for the first time in a MUMPP cohort — a quantitative, multivariate-adjusted estimate of the independent contribution of molecular resistance to fever trajectory (B = 20.85 hours per mutation-positive patient), advancing the field from descriptive resistance prevalence reporting toward molecularly informed outcome prediction. Third, the identification of lobar consolidation as an independent predictor of prolonged TTD exclusively in the multivariate model introduces chest imaging pattern as a novel, readily accessible clinical variable for risk stratification in MUMPP — a finding with immediate practical value in settings where genotyping is unavailable. Fourth, the structured three-month multi-organ safety surveillance — encompassing hematological, hepatic, renal, cardiac, and specialist dental assessments in all omadacycline-treated patients — provides the most comprehensive short-term organ-level safety dataset for a tetracycline-class antibiotic in this specific pediatric age group and infection context published to date, directly addressing the major safety concern that has historically impeded tetracycline use in school-age children.

This study also has several important limitations that must be considered when interpreting its findings. The retrospective, single-center, non-randomized design reflects real-world clinical decision-making but precludes causal inference and introduces potential for selection and performance bias. Because second-line treatment was allocated on the basis of clinician judgment and guardian consent rather than randomization, the groups were not randomly assigned: families who consented to omadacycline may have differed systematically from those who declined—for example, in health literacy, risk tolerance, or perception of illness severity—and inter-clinician variability in escalation thresholds may have further contributed to non-random allocation. This consent-based process also yielded a relatively small omadacycline group (n = 18), which limited statistical power and precluded formal adjustment by propensity-score methods. As a retrospective analysis based on medical-record review, the study is further subject to information bias and incomplete documentation rather than prospectively standardized data capture. Although baseline characteristics were well balanced between groups and 23S rRNA mutation status—a key potential confounder—was unavailable to clinicians at the time of treatment decisions, residual confounding from variables not captured in the clinical records cannot be excluded; these include the duration and adequacy of prior outpatient macrolide therapy, socioeconomic factors influencing healthcare-seeking behavior, and other unmeasured determinants of disease severity. An additional limitation concerns the choice of comparator: continued azithromycin after documented macrolide failure does not represent the most clinically relevant alternative treatment strategy according to current guidelines, which recommend doxycycline or fluoroquinolones. The observed efficacy advantage of omadacycline over continued azithromycin therefore cannot be extrapolated to superiority over these guideline-recommended agents. A further limitation concerns the corticosteroid use data: the significantly lower corticosteroid requirement observed in the omadacycline group (27.8% vs. 67.4%; *P* = 0.004) is a noteworthy finding, but its interpretation is constrained by the retrospective design, in which unmeasured differences in clinical trajectory between groups may have influenced steroid escalation decisions independently of antibiotic assignment. The three-month follow-up period, while sufficient to detect early post-treatment toxicity, is insufficient to capture delayed dental effects or assess long-term musculoskeletal safety; given that permanent tooth mineralization may extend beyond mid-adolescence in some individuals, longer observational periods are required before omadacycline can be unreservedly recommended in this age group. Additionally, the pediatric dosing regimen employed in this study was derived from body-surface-area scaling of the approved adult intravenous regimen without prospective pharmacokinetic validation in children, which introduces uncertainty regarding whether target drug exposures were reliably achieved across the range of body sizes and developmental stages represented in this cohort; dedicated pediatric pharmacokinetic studies are therefore an essential prerequisite before this BSA-based dosing approach can be broadly adopted. Finally, the single-center conduct of this study in a region with exceptionally high macrolide resistance rates may limit the generalizability of these findings to settings with different resistance prevalence, patient demographics, or institutional prescribing practices.

With regard to the multivariate regression model, several considerations regarding its stability and interpretive boundaries merit discussion. With 64 observations and 5 predictor terms (including 2 dummy variables for chest imaging), the ratio of observations to predictor terms is approximately 13 (64/5), which is at the lower end of conventional recommendations for stable linear regression estimates, suggesting that coefficient estimates may be sensitive to the specific sample composition. The model explains 51.0% of TTD variance (adjusted R² = 0.468), meaning that approximately half of the variability remains unexplained and is likely attributable to unmeasured factors such as *M. pneumoniae* bacterial load, host immunological response heterogeneity, timing and adequacy of antipyretic co-interventions, and inter-individual pharmacokinetic variability. The identification of lobar consolidation as a significant predictor only in the multivariate model (but not in univariate analysis) suggests the presence of confounding relationships among predictors; while this finding is clinically plausible, it should be interpreted with caution pending external validation in independent cohorts. Future studies with larger sample sizes should incorporate bootstrap stability analysis or cross-validation to assess the robustness of these predictor estimates.

These limitations collectively define a clear and actionable agenda for future research. Prospective randomized controlled trials directly comparing omadacycline with doxycycline and levofloxacin in adequately powered, multi-center cohorts are needed to establish the relative efficacy, corticosteroid-sparing effect, and cost-effectiveness of each agent within the therapeutic armamentarium for pediatric MUMPP. Dedicated pediatric pharmacokinetic studies incorporating population pharmacokinetic modeling are essential to define weight- and age-appropriate dosing regimens that reliably achieve pharmacodynamic targets while minimizing toxicity risk across different developmental stages. Longitudinal safety studies with systematic dental monitoring extending to the completion of permanent dentition, and incorporating validated patient-centered outcome instruments such as the Pediatric Quality of Life Inventory (PedsQL), will be required to provide a comprehensive assessment of the long-term safety and quality-of-life impact of omadacycline therapy in this population. Finally, future investigations should integrate dynamic markers of treatment response — including quantitative MP bacterial load kinetics, host-specific transcriptomic signatures, and serial inflammatory biomarker trajectories — to elucidate the mechanisms underlying differential fever resolution and to identify candidate biomarkers for early individualized treatment escalation in children with MUMPP.

### 4.6 Conclusions

In this retrospective cohort, second-line therapy with intravenous omadacycline was associated with significantly faster fever resolution than continued azithromycin treatment, with a reduction in TTD of approximately 22 hours and a significantly higher 72-hour defervescence rate. Omadacycline was additionally associated with a substantially lower requirement for systemic corticosteroids and demonstrated preliminary short-term safety data that require confirmation through larger studies with extended follow-up. Multivariate analysis identified continued macrolide therapy, 23S rRNA mutation positivity, systemic corticosteroid use, and lobar consolidation as independent predictors of prolonged defervescence, providing a basis for individualized risk stratification. While prospective randomized trials are needed to confirm these findings and establish long-term safety, omadacycline represents a promising candidate in the pediatric antimicrobial armamentarium for macrolide-unresponsive pneumonia.

## Data Availability

The datasets analyzed during the current study are not publicly available owing to privacy protection and ethical restrictions, as they contain identifiable patient information. Requests to access these datasets should be directed to Yao Sun, Department of Pediatrics, Nanjing Lishui People’s Hospital, Nanjing, China. Email: 545560404@qq.com.
